# Dogs' Expectation about Signalers' Body Size by Virtue of Their Growls

**DOI:** 10.1371/journal.pone.0015175

**Published:** 2010-12-15

**Authors:** Tamás Faragó, Péter Pongrácz, Ádám Miklósi, Ludwig Huber, Zsófia Virányi, Friederike Range

**Affiliations:** 1 Department of Ethology, Eötvös Loránd University, Budapest, Hungary; 2 Department of Cognitive Biology, University of Vienna, Vienna, Austria; 3 Clever Dog Lab, Vienna, Austria; CNRS - University Paul Sabatier, France

## Abstract

Several studies suggest that dogs, as well as primates, utilize a mental representation of the signaler after hearing its vocalization and can match this representation with other features provided by the visual modality. Recently it was found that a dogs' growl is context specific and contains information about the caller's body size. Whether dogs can use the encoded information is as yet unclear. In this experiment, we tested whether dogs can assess the size of another dog if they hear an agonistic growl paired with simultaneous video projection of two dog pictures. One of them matched the size of the growling dog, while the other one was either 30% larger or smaller. In control groups, noise, cat pictures or projections of geometric shapes (triangles) were used. The results showed that dogs look sooner and longer at the dog picture matching the size of the caller. No such preference was found with any of the control stimuli, suggesting that dogs have a mental representation of the caller when hearing its vocalization.

## Introduction

Several theoretical and field studies have shown that animals estimate the physical characteristics of the opponent before starting costly fights [1], [2]. Often such estimations are based on visual displays before starting a fight (e.g. red deer (*Cervus elaphus*): [3], elephant seals (*Mirounga angustriostris*): [4] and cichlid fish: [5]). However, ritualized displays of body size are often considered dishonest signals since the signalers may appear larger than they are (e.g. parallel walking of deer, fur erecting, spreading fins, erecting gills, etc. [6]).

During acoustic communication the parameters of vocal signals are affected by physical constraints, which are dependent on the anatomical features of the animals and cannot easily be manipulated, resulting in a reliable predictor of body size [7]. The physical constraints responsible for the reliability are described by the source-filter theory of voice production [8]. In brief, when the air from the lungs flows out between the two vocal folds, they start to resonate, which generates an acoustic signal (source). The resonance is defined as the fundamental frequency of the sound and dependent on the size and the tension of the vocal folds. When this signal passes through the supra-laryngeal vocal tract (the trachea, the oral and optionally the nasal cavity), the air also starts to resonate in this tube. Consequently, the natural resonances of this air column interfere with the signal's resonances and the vocal tract acts like a frequency band filter enhancing or extinguishing particular bands of the signal (filter). The enhanced bands then represent the formant frequencies of the signal. The length of the supra-laryngeal vocal tract, which is dependent on the body size of the caller, determines the frequencies of the formants and especially the spacing between them [9].

In human speech, the shape and change of the formant frequencies are responsible for the differentiation of speech sounds [10], but also carry indexical cues about the callers' sex and body size [11]. Early anatomical studies suggest that the manipulation of the vocal tracts' shape and size is uniquely human. However, Fitch and Reby (2001) [12] have shown that besides humans, several mammalian species, including pigs, goats, monkeys and dogs, are able to actively change the characteristics of their vocal tract during vocalization. When a species gains such a trait, it gives an opportunity for dishonest signaling, which can lead to an evolutionary arms race between signalers and receivers modifying their ability to precisely perceive the exact size of the caller [7]. For example, during roaring, male red deer can lower their larynx to lengthen their vocal tract, thus closing the formant frequencies in their signals which indicate bigger body size. For red deer, this and the correct perceived assessment of body size, is advantageous because females prefer larger body size of mates [13] and males use the formant spacing of the callers as a cue for body size during their agonistic encounters [14]. Both the size assessment of the males and the choice of the females put selection pressure on broadcasting of size information. The ability to lengthen the vocal tract however provides a way to lie about body size. In summation, perception of formants and formant spacing can be an important cue for judging size of conspecifics, as several studies have shown in humans [15], the rhesus monkey [16] and dogs [17], [18].

Dogs, like other species in the Canidae family, have a diverse vocal communication system [19] and recent studies have shown that dog barks have a communicative role in dog-human [20], [21] and dog-dog communication [22], [23]. Growls are also important for communication because they carry context specific information [24]. Moreover, Taylor and co-workers have shown that growls recorded in agonistic contexts carry indexical cues because formant spacing correlated with body size [18]. In addition, humans were sensitive to this indexical cue [18] and dogs showed a different pattern of explorative behavior in response to different formant-manipulated growls [25]. However, it is still unknown whether dogs are able to match size related acoustical information with visual size information.

In order to investigate this question, we used the preferential-looking paradigm, which has already been used successfully to test similar questions in rhesus monkeys [26]. The rationale of this paradigm is that individuals prefer to look at visual stimuli that correspond to broadcasted auditory stimuli, instead of non-matching visual stimuli (human infants: [27], [28]; capuchin monkeys: [29]; rhesus monkeys [30]). Therefore, in order to test if dogs could also match visual with acoustical information, we presented dogs with a choice of two pictures of the same dog, together with a playback of a growl recorded in a food competition context. One of the two pictures was manipulated to show smaller or larger body size compared to the growling dog, while the other picture was matched in size to the growling dog.

We expected, according to the other studies using the preferential-looking-time paradigm, that dogs would look longer at a picture of a dog that was matched to the signalers' body size and moreover, that no preference for either picture would be shown in control trials where either no size information was given in the acoustical domain (presentation of noise) or non-dog pictures were presented (shapes or cat pictures).

## Results

**Figure 1:**
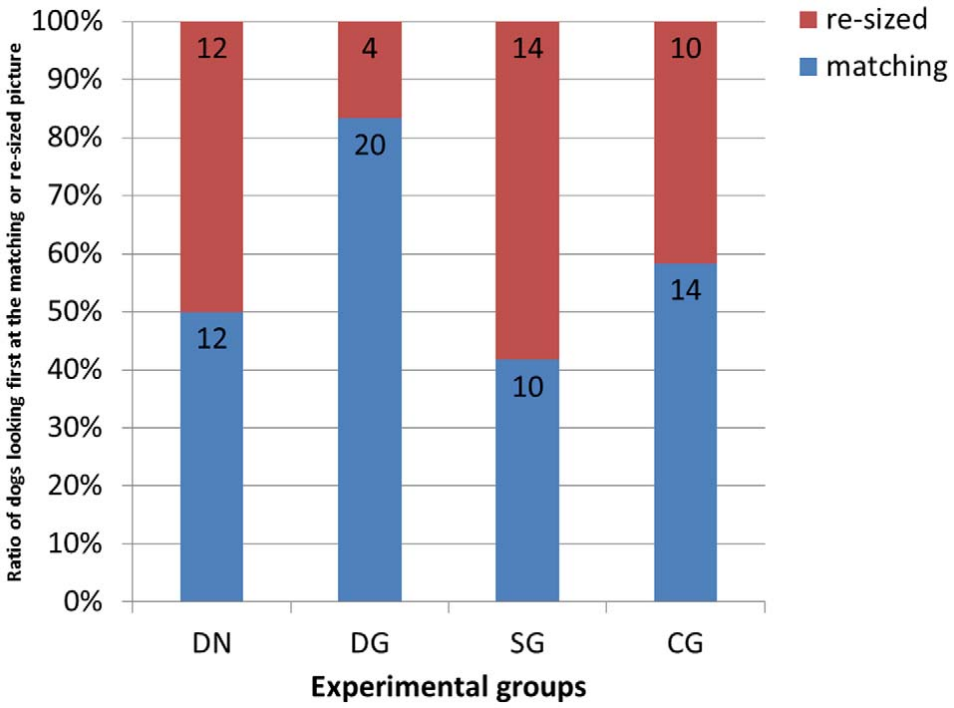
First glance at the pictures. This graph shows the number and ratio of dogs glancing first at the matching or at the resized picture in each group.

**Figure 2:**
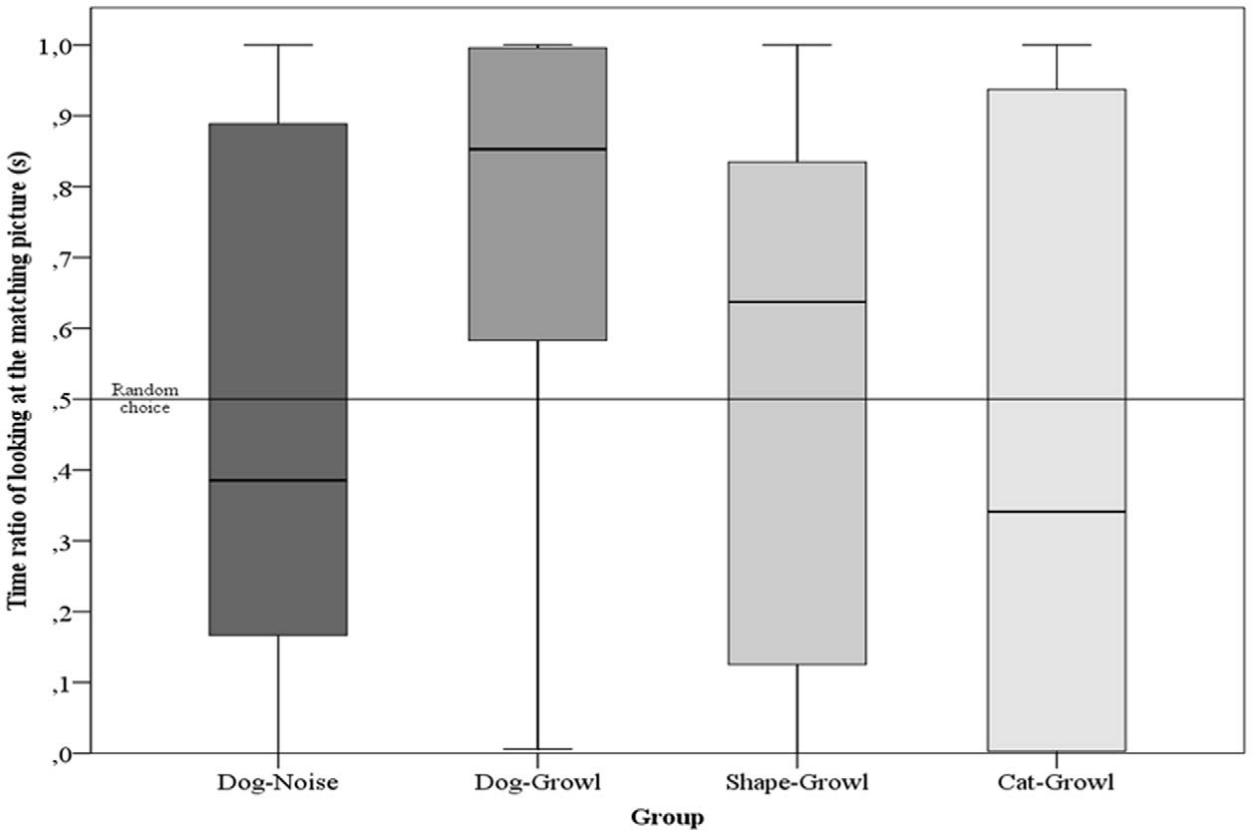
Looking preference towards matching picture. This graph shows the time ratio of looking at the matching sized picture of dogs in each group. The horizontal line at 0.5 represents random choice when dogs show no preference. The area above the line represents preference towards the matching sized picture. The boxplots shows the median, interquartiles and outliers.

The latency of looking at the matching and the resized picture showed that dogs in the Dog-Growl group looked earlier at the matching picture than at the non-matching picture. 20 of 24 subjects in this group looked first at the matching picture (two tailed Binomial test with 50% expected value: p = 0.0015), while in the other three groups we found no significant difference from the expected 50% (Binomial test: DN (50%): p = 1; SG (58.3%): p = 0.541; CG (41.7%): p = 0.541) (Figure 1). Moreover, the dogs in the test group also showed a strong looking preference towards the matching picture (Wilcoxon test: **p = 0.0022**) compared to the control groups (Wilcoxon test: DN: p = 0.9441; SG: p = 0.8334; CG: p = 0.8774) (Figure 2).

When comparing the behavior of the dogs in all of the experimental groups, we found that dogs spent more time looking at the stimuli when animal pictures were presented as compared to triangles (Kruskal-Wallis test: χ2 = 27.003; p<0.001; Dunn post hoc test: DN vs. DG: p>0.05; **DN vs. S: p<0.001**; DN vs. C: p>0.05;**DG vs. SG: p<0.01**; DG vs. CG: p>0.05; **SG vs. CG: p<0.001**), suggesting that the visual information conveyed by the pictures on natural objects was more interesting to the dogs than the artificial shapes.

None of the groups showed significant preference towards the bigger picture (Wilcoxon test: DN: p = 0.0738; DG: p = 0.8334; SG: p = 0.4389; CG: p = 0.4732). This suggests that the visual proximity did not affect the subjects' looking behavior. Moreover we tested if other factors had an effect on the looking behavior of the dogs, but neither the size of the growling dog (Mann-Whitney test: DN: Z = −0.696; p = 0.514; DG: Z = −0.262; p = 0.793 SG: Z = −1.049; p = 0.316 CG: only small dogs' growl were used in this group), the size of the non-matching picture (DN: Z = −1.827; p = 0.068; DG: Z = −0.145; p = 0.887 SG: Z = −0.586; p = 0.558 CG: Z = −0.614; p = 0.551), nor the size (DN: Z = −0.176; p = 0.86; DG: Z = −1.193; p = 0.233 SG: Z = −1.182; p = 0.237 CG: Z = −0.029; p = 0.977) or the sex of the subject (DN: Z = −0.090; p = 0.953; DG: Z = −0.088; p = 0.930 SG: Z = −0.952; p = 0.341 CG: Z = −1.026; p = 0.305) had an effect on the looking preference.

Another interesting pattern was found when we compared the side preference in the looking behavior of the dogs. In the cat-picture group a strong left gaze bias was found, while in the other three groups we found no such preference (Wilcoxon signed rank test: DN: p = 0.1140; DG: p = 0.5271; S: p = 0.2897; **C: p = 0.0087**).

## Discussion

The dogs' looking behavior suggests that they can extract size information encoded in the growls of conspecifics. When confronted with two dog pictures while hearing the growl, dogs looked sooner and longer at the picture showing a dog matched in size to the growling dog, than a picture of a smaller or larger one, which means that they linked the acoustic size cue with the visual information provided by the pictures. However, our subjects did not generalize the size cue to any type of objects e.g., they showed no looking preference if the growl playbacks were paired with non-dog visual stimuli such as triangles or cat pictures. The fact that dogs had a looking preference exclusively when the two modalities were both showing dogs, suggests that our subjects linked the growls with the dog pictures, further suggesting that dogs might be able to activate a specific mental representation of the signaler with respect to the species and the size.

Riede and Fitch (1999) [17] showed that the formant spacing in dog growls correlated not only with the vocal tract length, but also with the body size of the signaler. This suggests that formant dispersion can function as a reliable cue for dogs to assess the body size of conspecifics. Growls are mostly emitted as a threatening signal during agonistic social contexts, such as territory defense or food competition [19], [31], thus the ability to estimate body size and fighting ability based on these vocalizations could be advantageous for the receivers. As Taylor and co-workers (2008) [18] have shown, formant dispersion is an important clue for humans to assess the size of dogs when they growl. In addition, they modeled in a playback study an intrusion of a strange dog to the subject dogs' household and they found that the subjects behave differently according to the perceived intruders' body size. Large dogs showed more explorative behaviors when they heard growls in which the formant dispersion showed smaller dogs than their own size [25]. However, there was no other effect of the intruders' apparent body size when it was larger, or when the subjects were smaller. In contrast, our results showed that dogs are able to precisely match the size encoded in the growls with visual information about the emitter's size. Moreover, our findings strengthen the idea that looking preference in the test group was evoked by acoustic information provided by the growls. In contrast with Taylor and colleagues' [5]results, we did not find any effect of the size of neither the growling dog, nor our subjects. This was probably caused by the difference between the experimental environments used during the growl playbacks. While Taylor's study modeled an intrusion into the household of the subjects, in which a more active explorative behavior seems appropriate, our experiments were conducted in a strange place, where a more passive reaction can be expected from dogs.

Although we have little knowledge about how animals perceive the two dimensional representations of real world objects, numerous studies on animals and humans use pictures instead of real objects to investigate cognitive abilities [32]. Several studies have used picture presentations to test individual and species specific recognition (for example in sheep [33] and cattle [34]). In an earlier study, Adachi and co-workers demonstrated that dogs show a surprise reaction if a picture of a stranger's face coupled with the voice of their owner was shown to them, and similarly they looked longer at the owner's picture when a stranger's voice was played back [35]. Such intermodal matching ability has been shown in several other species recently in connection with various different cognitive abilities (individual recognition in a chimpanzee (*Pan troglodytes*): [36]; recognition of communicative signals in capuchin monkeys (*Cebus capucinus*): [29] facial expressions in rhesus monkeys (*Macaca mulatta*): [30] and size perception: [26]). Our results and the aforementioned studies, suggest that dogs as well as primates, possess the ability to have a mental representation of the signaler after hearing its vocalization and they can match this representation with other features provided by other modalities.

The overall duration dogs spent observing the pictures did not vary if they heard a growl or a noise and also did not differ between the groups that saw dog or cat pictures. However, the triangles grabbed the attention of the dogs markedly less. The reason for this can be that complex pictures might contain more processable information for dogs than triangles. There are some studies which suggest that dogs are capable of extracting some information from pictures. An earlier study reported that young and adult dogs, when presented with a life-size dog painting, sniffed those areas of the picture which are normally investigated by conspecifics during a first encounter [37]. Another study by Pongrácz and co-workers, investigated dogs' performance in a pointing and an obedience task and they found that when comparing real and live video projected signaling of the owner, the dogs readily obeyed the life size two-dimensional projections [38]. Another more recent work showed the discriminative capability of dogs with pictures of dog and human faces, suggesting that dogs can use facial cues to differentiate individual dogs and humans [39]. Moreover, Range and co-workers showed that during a touch-screen based procedure, dogs were able to classify photographs of dogs and landscapes correctly by perceptional differences [40]. Fagot and co-workers differentiated three levels of pictorial processing: independence, confusion and equivalence [41]. In independence, the subject makes no connection between the picture and its referent, while equivalence means that the animal perceived the picture as a representation of a real object. On the lowest level, the viewer of the picture confuses it with its content and acts like it would be the real three-dimensional object. We assumed that the dogs in our study worked on this lowest level in order to link the visual and acoustical modalities. The dogs in our study showed significantly more attention towards the pictures if the pictures showed dogs or cats. Moreover, the fact that the dogs showed a preference towards the matching sized picture, only on the occasion of dog-growl intermodal pairings, suggests that they could perceive the dog pictures as dogs. However to confirm this, other more specific tests would be necessary, such as showing pictures of different species pairs, such as dogs versus cats, while playing dog or cat specific vocalizations. We also realize that it is possible that the longer looking times at the dog and cat pictures can be attributed to different causes. Looking at dog pictures can be an attentive response, elicited by perceiving conspecifics, while the cat picture in odd pairing with dog growls, can cause a surprise response with orientation behavior. This is supported by the fact that dogs showed marked left gaze bias only when looking at the cat pictures. This interesting result can be explained by the asymmetric manner of perception of sensory stimuli. Recent studies showed a left gaze bias in dogs in the case of simultaneous bilateral heterospecific visual presentations (cat and snake silhouettes), [42] as well as acoustical [43] unanimated natural sound stimuli (thunder). More importantly, when dogs were presented with a dog silhouette or dog vocalizations, they showed no bias or a right gaze bias in the above mentioned experiments, suggesting that similarly as in other mammals and humans, the right hemisphere shows increased activity in association with novel, attention grabbing or unexpected stimuli [44]. Because the auditory tract fully crosses in the dogs' brain and the optical nerve crosses 75% as well [45], the left gaze bias towards cat pictures, coupled with growls found in our study, could be explained by the non-matching visual and acoustical modalities functioning as a novel, non-expected stimulus that is processed more by the right hemisphere causing a left gaze bias.

The lack of a right gaze bias in our study, when compared to the study by Siniscalchi and co-workers' experiments where dogs were presented with conspecific vocalizations [42], [43], can be explained by the fact that our sound playbacks came from the middle, rather than the bilateral presentation of Siniscalchi's experiments. It is also possible that our experimental manipulation masked out this gaze effect because the size information might have had a stronger impact on the looking behavior of the dogs.

In conclusion, we have shown for the first time that dogs can match cross-modal information between pictures and sounds and we provided evidence that dogs can assess accurately the size of a growling dog based on the acoustic information. Moreover, our results suggest that dogs are able to perceive species specific information based on pictures.

## Materials and Methods

### Ethics statement

The owners and their dogs participated in this study voluntarily. The owners were present at the behavioral testing. The testing procedure was short and entirely non-invasive.

No special permission for the use of animals (dogs) in socio-cognitive research such as this study is required in Hungary or Austria. The relevant committees that allow research without special permission in regard to using animals are the University Institutional Animal Care and Use Committee (Hungary) and Tierversuchskomission und Bundesministerium für Wissenschaft und Forschung (Austria).

### Subjects

Participants in our study were well socialized family dogs, recruited from the database of the Clever Dog Lab, Vienna. In total, 116 dogs participated in this study. Of these, 20 dogs had to be excluded from the sample due to technical problems (n = 11) and behavioral difficulties (n = 9), leaving a total of 96 dogs (male: 42, female: 56, mean age: 4.7±2.6 years) for analyses.

### Experimental design

We used four different types of stimuli pairs during the experiment in a parallel intermodal presentation (see [46]):

Dog-Growl (DG) – matching modalities: presentation of dog growls with projection of dog pictures. (N = 24)

Dog-Noise (DN) – non-informative sound: presentation of Brownian noise with projections of dog pictures. This control was conducted to test if there was any effect caused by the size difference of the dog pictures on the looking behavior of the subjects. (N = 24)

Shape-Growl (SG) – non-informative picture: presentation of dog growls with projection of triangles. This control group was used to test if the looking preferences of the dogs were nonspecific in that they would occur also with non-natural, unknown pictures. (N = 24)

Cat-growl (CG) – non-matching modalities: presentation of dog growls with projection of cat pictures. This control was conducted to test the effect of the nature of the pictures. (N = 24)

These latter two controls were specifically designed to test if possible positive results of the test condition was dependent on the nature of the visual stimuli or not. We tested 24 subjects in each of the four experimental groups. All dogs tested in the first three groups were familiar with other dogs. Moreover, subjects in the last group were also familiar with cats (once lived, currently living together or in regular contact with cats).

We used a between-subject design to avoid order effects and possible habituation of the subjects to the stimuli. Thus, each dog was tested only once with one sound sample paired with a visual stimuli.

### Stimuli design

**Figure 3:**
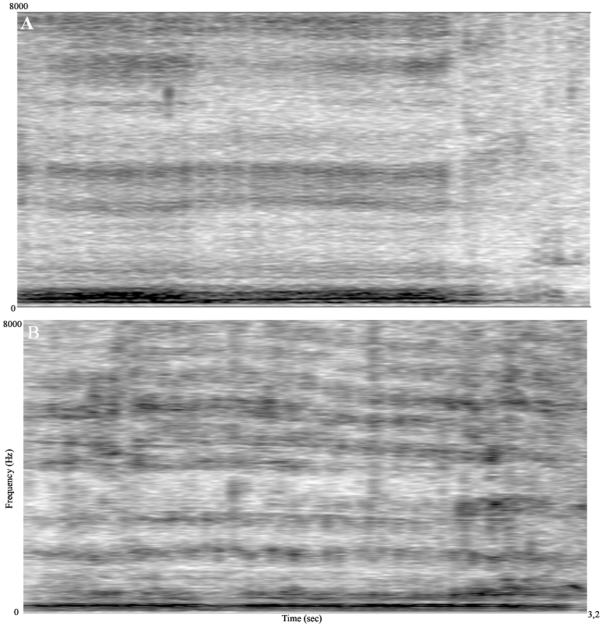
Spectrogram examples of the small and large dogs' growl. A. Small dog's growl (weight: 8,5 kg, height: 32 cm, F0: 131 Hz, dF: 2950 Hz) B. Large dog's growl (weight: 34 kg, height: 64 cm, F0: 63 Hz, dF: 764 Hz). The fundamental frequency is shown as the lowest dark line in the spectrum, while the formant frequencies are the broader, parallel dark stripes in the upper area. It is easy to see that the small dog's growls have a higher F0 and there are wider frequency steps between its formant frequencies.

The auditory stimuli were chosen from a pool of pre-recorded food guarding growls from 12 different dogs and previously generated Brownian noise (Audio S1) samples (Adobe audition 1.5). We used a food guarding growl because these growls are used against other conspecifics in an agonistic encounter (for detailed information about the growls and how they were collected see [24]). The physical parameters (height at withers, weight) of the twelve growling dogs were known (see Table S1). We used the growls of two groups of markedly different sized dogs (six “small dogs”: shorter than 52 cm and six “large dogs”: taller than 60 cm at the withers) for the experiments (Figure 3, Audio S2 and S3). The growls in the two size categories differed significantly in their acoustic structure (parameters were measured with PRAAT, for exact definition of these acoustic parameters see [17]). The formant dispersion, as mentioned in our introduction, is in strong negative correlation with body size, thereby acting as a reliable cue. The same was true in our growl samples: small dog growls had significantly higher formant dispersion than the large growls (mean dF - small: 2658±232 Hz; large: 671±253 Hz; unpaired t-test: t = 14.17; p<0.0001). The fundamental frequency in most of the examined species shows only weak or no correlation with body size because the anatomy of the vocal folds is not affected by the skeletal and muscular conformation of the body [7]. However, different dog breeds show such high morphological diversity, that their vocal fold size and fundamental frequency can also correlate with the physical parameters of the body [18]. The two subsets of growls used in this study also differed in their fundamental frequencies: small dogs' growls had significantly higher fundamental frequency than the growls of the large dogs (mean F0 - small: 130±25 Hz; large: 90±253 Hz; unpaired t-test: t = 2.83; p = 0.018) suggesting that the two acoustical parameters together can act as an indexical cue for the dogs in our study.

The visual stimuli were digital photos of twelve different shortnosed dog breeds (DG, DN), twelve different cat breeds (CG) and twelve differently colored triangle shapes with various internal angles (SG) (for some examples see Figure S1). The pictures were presented in front of a homogenous black background. In each experimental trial, we showed the same two pictures, but one was adjusted to be life sized (the size of the projection was similar to the size of the actual growling dog - matching), while the other picture was 30% smaller or larger than the matching picture (resized). The projections' height was measured on the canvas from the ground to the withers in the case of dogs (22−83 cm) and cats (22−67 cm). The length of the vertical side was used for the height of the triangles.

Within each group half of the subjects heard a “small dog” growl, and the other half heard a “large dog” growl. The side where the manipulated picture appeared was balanced within each group. Each growl was presented with the same pictures to two different dogs, but the size was differently adjusted: one dog saw a smaller picture paired with the matching sized photo, while the other one saw a larger picture paired with the matching sized photo. Thus, each growl was used two times for playbacks within each group, except in the Cat-Growl group. In the CG group we used only the six small dogs' growls, as cat pictures adjusted to match their size to the large dog growls would be unnaturally big. Thus, in the Cat group, each growl sample was used in sum four times, twice-twice with two different cat pictures (for the detailed set see Table S1).

### Experimental set-up

A chair for the dog's owner was placed at one side of the darkened experimental room (5 m×6 m) facing the canvas on which the pictures will be presented to the dog (Figure S2). During the entire experiment, the owner was listening to music through headphones to prevent him/her from hearing the playbacks and influencing the behavior of the dog unintentionally.

The dog sat or laid between the owners legs, also facing towards the canvas (size: 2.3 m×1 m) on which the two pictures will be shown (height of 30−95 cm depending on the size of the growling dog, 2.2 m apart). The pictures were presented by a projector which was positioned behind the owner at a height of 1.5 m. The speaker was placed on the ground in front of the canvas in the middle of the two presented pictures. We used four cameras to record the behavioral response of the dog. The first camera was put next to the projector (Cam1) recording the projection on the canvas. The second camera (Cam 2) – a zero lux camera that could record in low light density – was put in front of the speaker to record the dogs' response. Near the owner's chair, an infrared lamp was directed to the dog's face in order to lighten them up for the zero lux camera, without interfering with the projections. Finally, two wide-angle cameras with high light sensitivity recorded the events in the entire room (Cam3 and 4). We used Cam1's microphone for sound recording. For effective projection of pictures, the lights were switched off and the windows were covered with a curtain. The experimenter controlled the events from the neighboring room via a closed-circuit video system and a PC was used for the picture presentation, audio play back and video recording.

The stimuli were displayed during the experiments as PowerPoint slideshows. Three slides were used: the first and the last were homogenous black, while the middle slide contained the pictures and the sound. The change between the first and second slide (stimuli presentation) was controlled by the experimenter, and was dependent on the behavior and attention of the subject (see below for the exact criteria) while the sound sample was automatically played as soon as the visual stimuli appeared. The change between the second and third slides (disappearance of the pictures) was automatic after 20 seconds. The volume of the different growls was measured from the location where the dogs sat and adjusted to the same level (65 dB) prior to the experimental trials.

### Procedure

Before the experiment, the owner was informed about the procedure and told his/her tasks during the experiment, although no detailed information such as the type of given stimuli was explained. Next, the dog and its owner entered the experimental room and the dog was allowed to explore the unfamiliar room for approximately 2 minutes. During the experiment, the owner was asked to sit on the chair with headphones on and listen to music on an mp3 player. The volume of the music was adjusted to a level that prevented the owner from hearing the sound playbacks.

The dog was sitting or lying in front of the owner on the floor, facing the screen (beginning body posture). The dog was not on leash and the owner was allowed to position it with their hands just before the appearance of the pictures. The experimenter switched off the lights and asked the owner to adjust the dog gently into the beginning body posture. Then he zoomed Cam2 at the dogs' face and started the video recording before finally leaving the room. During these events the first black slide was projected at the canvas.

After the experimenter left and the door was closed, the dog was in the beginning body posture for at least 10 seconds and the sagittal axis of the dog's head pointed to the center of the canvas (looking to the middle), the experimenter then switched to the second slide which activated the growl. The projected pictures then appeared on the canvas at the two lateral parts of the lower side of the canvas for 20 seconds. During the projection of pictures, the owners were not allowed to look at the pictures and they were not allowed to talk or touch their dog. If the owner interacted with the dog during the projections, the data were not used for the analysis. After the projection of the pictures ended, the experimenter entered the room and the experiment was finished. If the dog did not orient towards the canvas during the growl playback, or did not look at either of the pictures, the trial was considered to have failed and the data of the subject were not used for the analyses.

### Behavior analyses

**Figure 4:**
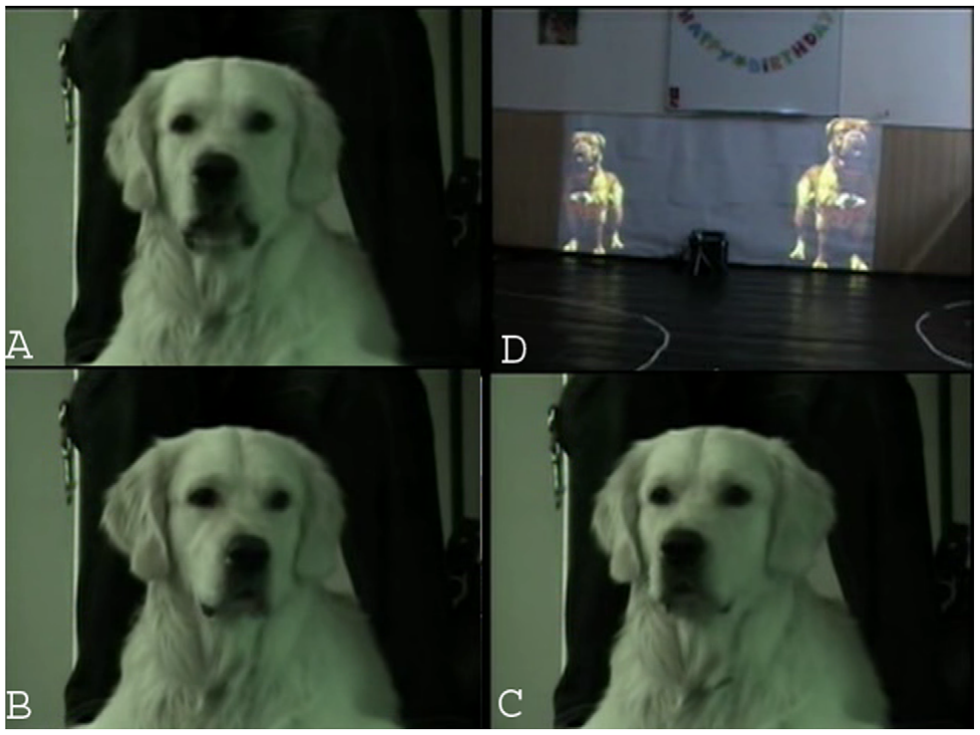
Examples of the looking behavior. A, Subject looking at the middle. B, Subject looking at the left picture. C, Subject looking at the right picture. D, Projections of dog pictures.

We measured the looking preference of the dogs. Their orienting behavior and the time of looking at the two pictures during the projections were blind coded by an assistant (Nándor Takács) with a Solomon Coder (behavior coding software developed by András Péter, Dept. of Ethology, Budapest). Inter observer reliability was tested on a random subsample (10 dogs from each group) of recordings (average Cohen Kappa: 0.87). Orientation towards one side was defined as the angle between the sagittal axis of the dogs' head and the center of one of the pictures, which is less than 10 degrees (looking at the picture), and the dogs' eyes fixed on that projected picture (Figure 4 and Video S1 – for annotated version see: http://www.cmdbase.org/web/guest/play/-/videoplayer/9). We coded the behavior from the beginning of the projection until the pictures disappeared to measure the latencies and the looking time, the maximum of these time variables was 20 seconds. We transformed the latency data to a binary variable to analyze which picture the subject glanced at first (1 – looking first at matching picture, 0 – looking at resized picture). We calculated the overall looking time (the sum of time looking at any of the two pictures) and the time ratio of looking at the matching picture (time of looking at one given picture divided with the overall looking time). The ratio of looking at the bigger picture and at the left picture was also calculated to test the possible effect of the size and the position of the picture independently from the acoustic stimuli within groups.

### Statistical analysis

None of our behavioral variables were normally distributed, thus we used non-parametric statistical tests. To test within-group effects, we used exact Binomial and Wilcoxon matched-pair tests, for between group comparisons we used Kruskal-Wallis tests. To measure looking and side preferences, the looking time of the groups was compared to a hypothetical 0.5 median (no preference) with Wilcoxon signed-rank tests. All tests were performed with SPSS 15, except the looking preference tests and the Dunn post hoc tests, for these GraphPad Instat statistical software was used. For correcting on multiple measures, we applied FDR correction.

## Supporting Information

Figure S1 **Examples of the used visual stimuli.**
(TIF)

Figure S2 **Arrangement of the experimental room.**
(TIF)

Table S1 **Data of the subjects, specifications of the growl samples and the experimental arrangement.**
(XLSX)

Video S1 **Video sample of the experiment.** The subject heard a big dog's growl, the matching picture was the left one (from the view of the dog), while the modified picture was bigger than the actual growling dog. The subject looked first and markedly longer at the matching picture, while observed the modified one for just a couple of seconds. (for annotated version see: http://www.cmdbase.org/web/guest/play/-/videoplayer/9)
(AVI)

Audio S1 **Sound sample of the used Brownian noise.**
(WAV)

Audio S2 **Sound sample of the used growls.** Small dog.
(WAV)

Audio S3 **Sound sample of the used growls.** Large dog.
(WAV)

## References

[1] Arnott G, Elwood RW (2009). Assessment of fighting ability in animal contests.. Anim Behav.

[2] Enquist M, Leimar O (1983). Evolution of fighting behaviour: Decision rules and assessment of relative strength.. J Theor Biol.

[3] Clutton-Brock TH, Albon S, Gibson R, Guinness F (1979). The logical stag: Adaptive aspects of fighting in red deer (L.).. Anim Behav.

[4] Cox CR (1981). Agonistic Encounters Among Male Elephant Seals: Frequency, Context, and the Role of Female Preference.. Integr Comp Biol.

[5] Enquist M, Leimar O, Ljungberg T, Mallner Y, Segerdahl N (1990). A test of the sequential assessment game: fighting in the cichlid fish Nannacara anomala.. Anim Behav.

[6] Maynard-Smith J, Harper D, Harvey PH, May RM (2003). Animal signals..

[7] Fitch WT, Hauser MD, Simmons AM, Popper AN, Fay RR (2003). Unpacking “Honesty”:Vertebrate Vocal Production and the Evolution of Acoustic Signals.. Acoustic communication.

[8] Fant G (1960). Acoustic theory of speech production.. Mouton De Gruyter.

[9] Fitch WT (2000). The phonetic potential of nonhuman vocal tracts: Comparative cineradiographic observations of vocalizing animals.. Phonetica.

[10] Lieberman PH, Blumstein S (1988). Speech physiology, speech perception, and acoustic phonetics..

[11] Smith DR, Patterson RD (2005). The interaction of glottal-pulse rate and vocal-tract length in judgements of speaker size, sex, and age.. J Acoust Soc Am.

[12] Fitch WT, Reby D (2001). The descended larynx is not uniquely human.. P Roy Soc B-Biol Sci.

[13] Charlton BD, Reby D, McComb K (2007). Female red deer prefer the roars of larger males.. Biol Letters.

[14] Reby D, McComb K, Cargnelutti B, Darwin C, Fitch WT (2005). Red deer stags use formants as assessment cues during intrasexual agonistic interactions.. P Roy Soc B-Biol Sci.

[15] Feinberg DR, Jones BC, Little AC, Burt DM, Perrett DI (2005). Manipulations of fundamental and formant frequencies influence the attractiveness of human male voices.. Anim Behav.

[16] Fitch WT (1997). Vocal tract length and formant frequency dispersion correlate with body size in rhesus macaques.. J Acoust Soc Am.

[17] Riede T, Fitch WT (1999). Vocal tract length and acoustics of vocalization in the domestic dog (Canis familiaris).. J Exp Biol.

[18] Taylor AM, Reby D, McComb K (2008). Human listeners attend to size information in domestic dog growls.. J Acoust Soc Am.

[19] Cohen JA, Fox MW (1976). Vocalizations in wild canids and possible effects of domestication.. Behav Proc.

[20] Pongrácz P, Molnár C, Miklósi Á, Csányi V (2005). Human listeners are able to classify dog (Canis familiaris) barks recorded in different situations.. J Comp Psychol.

[21] Pongrácz P, Molnár C, Miklósi Á (2006). Acoustic parameters of dog barks carry emotional information for humans.. Appl Anim Behav Sci.

[22] Maros K, Pongrácz P, Bárdos G, Molnár C, Faragó T (2008). Dogs can discriminate barks from different situations.. Appl Anim Behav Sci.

[23] Molnár C, Pongrácz P, Faragó T, Dóka A, Miklósi Á (2009). Dogs discriminate between barks: the effect of context and identity of the caller.. Behav Proc.

[24] Faragó T, Pongrácz P, Range F, Virányi Z, Miklósi Á (2010). ‘The bone is mine’: affective and referential aspects of dog growls.. Anim Behav.

[25] Taylor AM, Reby D, McComb K (2010). Size communication in domestic dog, Canis familiaris, growls.. Anim Behav:.

[26] Ghazanfar AA, Turesson HK, Maier JX, van Dinther R, Patterson RD (2007). Vocal-tract resonances as indexical cues in rhesus monkeys.. Curr Biol.

[27] Soken NH, Pick AD (1999). Infants' perception of dynamic affective expressions: do infants distinguish specific expressions?. Child Dev.

[28] Patterson ML, Werker JF (2002). Infants' ability to match dynamic phonetic and gender information in the face and voice.. J Exp Child Psychol.

[29] Evans TA, Howell S, Westergaard GC (2005). Auditory–isual cross-modal perception of communicative stimuli in tufted capuchin monkeys (Cebus apella).. J Exp Psychol Anim B.

[30] Ghazanfar AA, Logothetis NK (2003). Facial expressions linked to monkey calls.. Nature.

[31] Yeon S (2007). The vocal communication of canines.. J Vet Behav.

[32] Bovet D, Vauclair J (2000). Picture recognition in animals and humans.. Behav Brain Res.

[33] Bouissou MF, Porter RH, Boyle L, Ferreira G (1996). Influence of a conspecific image of own vs. different breed on fear reactions of ewes.. Behav Proc.

[34] Coulon M, Deputte BL, Heyman Y, Delatouce L, Richard C (2007). Visual discrimination by heifers (Bos taurus) of their own species.. J Comp Psychol.

[35] Adachi I, Kuwahata H, Fujita K (2007). Dogs recall their owner's face upon hearing the owner's voice.. Anim Cogn.

[36] Martinez L, Matsuzawa T (2009). Auditory-visual intermodal matching based on individual recognition in a chimpanzee (Pan troglodytes).. Anim Cogn.

[37] Fox MW (1971). Socio-infantile and Socio-sexual signals in Canids: a Comparative and Ontogenetic Study.. Z Tierpsychol.

[38] Pongrácz P, Miklósi Á, Dóka A, Csányi V (2003). Successful application of video-projected human images for signalling to dogs.. Ethology.

[39] Racca A, Amadei E, Ligout S, Guo K, Meints K (2009). Discrimination of human and dog faces and inversion responses in domestic dogs (Canis familiaris).. Anim Cogn.

[40] Range F, Aust U, Steurer M, Huber L (2008). Visual categorization of natural stimuli by domestic dogs.. Anim Cogn.

[41] Fagot J, Martin-Malivel J, Dépy D, Fagot J (2000). What is the evidence for an equivalence between objects and pictures in birds and nonhuman primates.. Picture perception in animals.

[42] Siniscalchi M, Sasso R, Pepe AM, Vallortigara G, Quaranta A (2010). Dogs turn left to emotional stimuli.. Behav Brain Res.

[43] Siniscalchi M, Quaranta A, Rogers LJ (2008). Hemispheric specialization in dogs for processing different acoustic stimuli.. PloS ONE.

[44] Andrew RJ, Watkins JA, Rogers LJ, Andrew  RJ (2002). Evidence for cerebral lateralization from senses other than vision.. Comparative Vertebrate Lateralization.

[45] Fogle B (1992). The dog's mind..

[46] Guihou A, Vauclair J (2008). Intermodal matching of vision and audition in infancy: A proposal for a new taxonomy.. Eur J Dev Psychol.

