# Supplementary figures and images for: Dogs' Expectation about Signalers' Body Size by Virtue of Their Growls
Source: PLoS One. 2010 Dec 15;5(12):e15175. doi: 10.1371/journal.pone.0015175 (PMC3002277; doi:10.1371/journal.pone.0015175)

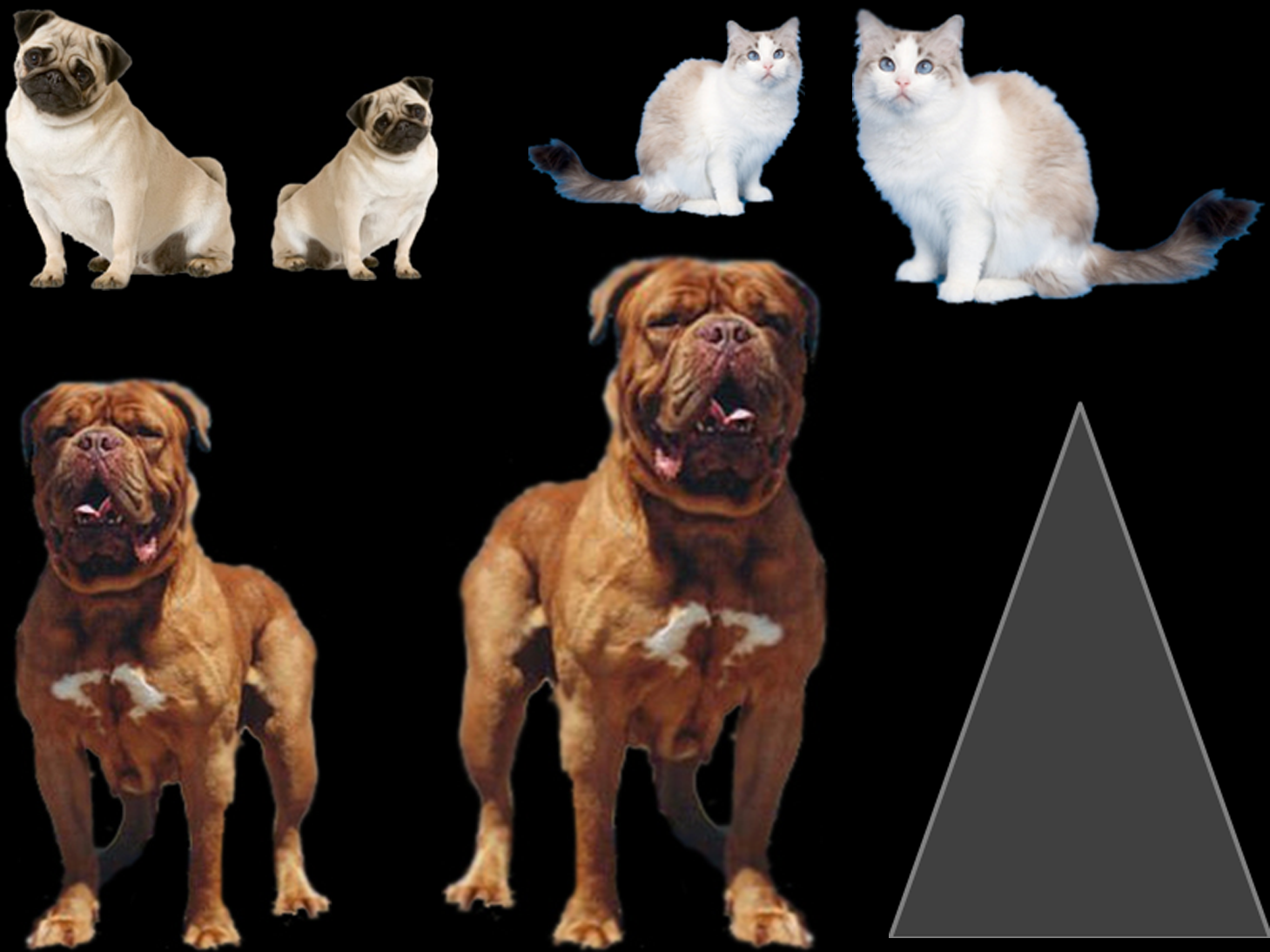

Supplement: Figure S1 — Examples of the used visual stimuli. (TIF) [file pone.0015175.s001.tif]

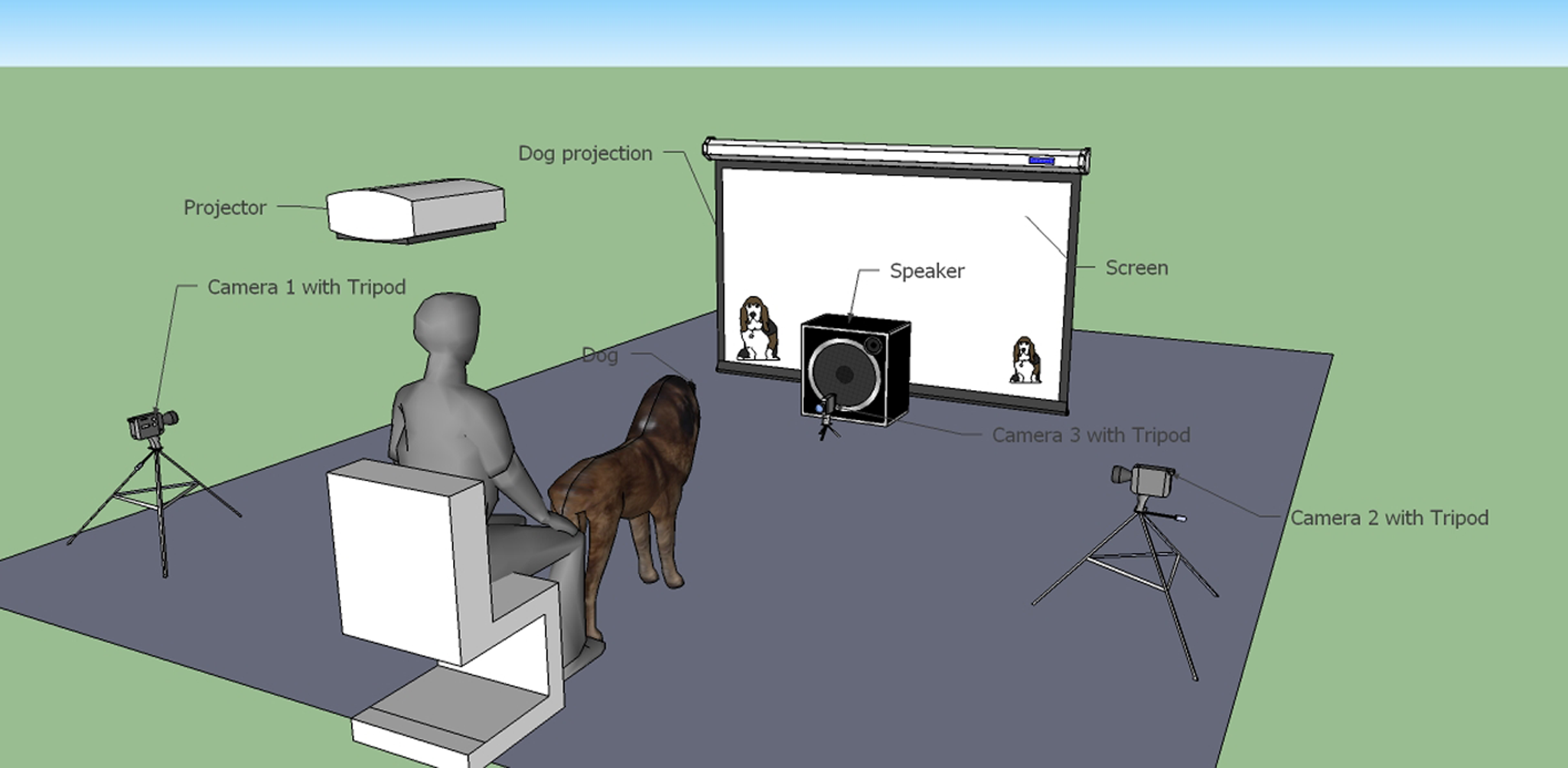

Supplement: Figure S2 — Arrangement of the experimental room. (TIF) [file pone.0015175.s002.tif]
